# Immunomodulating Effects of Heat-Killed *Lactobacillus rhamnosus* and *Lactobacillus reuteri* on Peripheral Blood Mononuclear Cells from Healthy Dogs

**DOI:** 10.3390/vetsci12030226

**Published:** 2025-03-02

**Authors:** Marie Cauquil, Thierry Olivry

**Affiliations:** 1 Nextmune, 3 rue Pierre-Gilles de Gennes, ZA Le Causse, 81100 Castres, France; 2 Nextmune AB, Riddargatan 19, 114 57 Stockholm, Sweden

**Keywords:** atopic dermatitis, postbiotics, immunomodulation, cytokines, *Lactobacillus*, dog, heat-killed bacteria, Tyndallized

## Abstract

Canine atopic dermatitis (AD) is a T-cell-driven inflammatory skin disease, characterized by an imbalance between the Th1 and Th2 immune responses. Probiotics (live bacteria) and postbiotics (inactivated, killed bacteria) have garnered attention for the management of AD in humans and dogs. Both probiotics and postbiotics possess immunomodulating properties that could be beneficial for allergic patients. This study aims to evaluate the immunomodulating effects of Tyndallized (heat-killed) postbiotics of *Lactobacillus rhamnosus* and *Lactobacillus reuteri,* which are active components of the Linkskin products (Nextmune, Palazzo Pignano, Cremona, Italy). Peripheral blood mononuclear cells (PBMCs) were isolated from healthy dogs and incubated separately with each postbiotic. The cytokine levels in the supernatants were measured before and after 12, 24, 48, and 72 h of incubation. Both Tyndallized lactobacilli significantly increased the levels of IL-12 and IFN-γ (Th1 cytokines) and IL-10 (associated with T regulatory cells), while the levels of the Th2 cytokine IL-4 remained stable. Overall, these two *Lactobacillus* postbiotics stimulated canine PBMCs to produce a cytokine profile typically associated with an anti-allergic response. Further studies are needed to evaluate the benefit of these postbiotics as an adjuvant for the reactive treatment or for the prevention of relapses of allergic flares in atopic dogs.

## Simple Summary

Atopic dermatitis is a common skin disease of humans and dogs that requires lifelong multimodal management. For several years, interest has increased in the oral intake of probiotics (live bacteria) to help prevent or control clinical signs of this disease. More recently, postbiotics (i.e., inactive, killed bacteria) have also been studied. The effects of probiotics and postbiotics on the immune system could be of interest in dogs with atopic dermatitis, a chronic inflammatory skin disease that involves an imbalance between the Th1 anti-allergic and Th2 pro-allergic immune responses. In this study, lymphocyte-rich white blood cells from healthy dogs were exposed to postbiotics of *Lactobacillus rhamnosus* and *Lactobacillus reuteri*, which are active components of the Linkskin products (Nextmune, Palazzo Pignano, Cremona, Italy). We found that these postbiotics enhanced the production of Th1 cytokines, such as IL-12 and IFN-γ, as well as the regulatory cytokine IL-10, while the influence on the Th2 cytokine IL-4 was minimal. Overall, the outcomes suggest that these postbiotics modulate the immune response in a way that could be helpful for the treatment or prevention of relapses of allergic diseases in dogs.

## 1. Introduction

Canine atopic dermatitis (AD) is a hereditary, typically pruritic, and predominantly T-cell-driven inflammatory skin disease [1]. In the acute stages of canine AD, a Th2 response seems to predominate, whereas in the chronic stages, immune responses seem to be mixed, with an interplay between multiple cytokines, including those typical of Th1 and Th17 immune responses [2,3,4]. Th2 cytokines promote the production of immunoglobulin E (IgE) antibodies by Bcells and favor the recruitment of inflammatory cells typically associated with hypersensitivity reactions, such as eosinophils.

As this clinical syndrome also involves an interplay between skin barrier abnormalities, allergen sensitization, and microbial dysbiosis [1], its management requires a multifaceted approach, including not only anti-inflammatory and anti-pruritic therapies but also allergen avoidance and prevention of relapses [5].

In recent years, research on the potential benefits of orally administered probiotics—live microorganisms that, when administered in adequate amounts, confer a health benefit on the host [6]—to patients with AD has gained interest. The concept of an oral intake of live probiotics in atopic subjects is grounded in the dogma that, in addition to the skin dysbiosis demonstrated in human [7,8] and canine [9,10,11] atopic patients, gut dysbiosis is also observed [7,8,9,12,13,14]. While the precise mechanisms explaining the link between intestinal dysbiosis and AD remain to be unraveled, it has been hypothesized that gut microbial dysbiosis could impact the skin and predispose to the development of AD through immunologic, neuroendocrine, and metabolic pathways [7,8,15,16].

*Lactobacillus* species and their strains are among the most investigated types and are commonly found in oral probiotic supplements. While some studies showed that probiotics have a preventive effect against childhood AD development and can alleviate symptoms in infants or adults, conflicting results also exist [8,17]. In dogs, three studies evaluated the benefit of live probiotics in treating signs of canine AD. The administration of *Lactobacillus sakei* for 8 weeks resulted in a reduction in CADESI-03 skin lesion scores; still, it did not improve the pruritus (PVAS) values compared to those of the control group at the study’s end [18]. In another study, *Lactobacillus paracasei* K71 was administered for 12 weeks to atopic dogs, which led to reductions in the CADESI and pruritus scores compared to baseline, but not to significant differences compared to the values of the control (antihistamine-treated) group. However, a significant reduction in the medication score was noted in the probiotic-treated compared to the control group [19]. Finally, another species of probiotics, *Enterococcus faecium*, was evaluated in atopic dogs, with no significant difference being observed in the dose reduction of oclacitinib compared to that of the placebo group after 12 weeks of administration [20].

Interestingly, the beneficial effects attributed to probiotics could come not only from the live bacteria themselves but also from other microbial structural or metabolic components [21]. In the past decade, inactivated, killed probiotic bacteria have gained interest, with some studies revealing their potential health benefits [21,22,23]. The International Scientific Association of Probiotics and Prebiotics coined the term “postbiotic” to designate a “preparation of inanimate microorganisms and/or their components that confers a health benefit on the host” [23]. Postbiotic bacteria are thus deliberately inactivated to terminate their viability using either heat, radiation, high pressure, or lysis. This can leave intact inanimate cells, cell components, or both [24]. One of the main advantages of postbiotics is their inherently higher stability than that of probiotics, which are challenging to keep alive. Postbiotics may exert their beneficial effect through a variety of mechanisms that include, among others, an enhancement of the gut epithelial barrier function and a modulation of local and/or systemic immune responses [21,23]. The immunomodulating properties of postbiotics are of particular interest in the case of AD, as they have the ability to commute a Th2 into Th1 and/or Treg responses [25,26,27]. However, the bacterial species, the method of bacterial inactivation, the immunological target, or the model used can all impact study results, thus preventing the comparison between experiments and making it difficult to generalize study findings [25].

In this study, we explored the immunomodulatory effects of heat-inactivated (Tyndallized) *Lactobacillus rhamnosus* SGL01 and *Lactobacillus reuteri* SGL06 on peripheral blood mononuclear cells (PBMCs) isolated from healthy dogs. Our objective was to assess how a co-culture of canine PBMCs with each of the two postbiotics influenced the secretion of representative Th1, Th2, and regulatory T cell (Treg) cytokines.

## 2. Materials and Methods

### 2.1. Animals

Blood from five healthy dogs without a history of allergy or digestive signs was collected in EDTA tubes. The samples were collected at the time of vaccination or routine spay/neuter after verbal consent from the owner was obtained. The dogs had not received any immunomodulatory drugs (e.g., ciclosporin, oclacitinib, or oral or injectable glucocorticoids) in the month preceding sample collection.

### 2.2. Peripheral Blood Mononuclear Cell Isolation

Peripheral blood mononuclear cells (PBMCs) were isolated using density gradient separation using Histopaque 1077 Hybri-Max (Sigma-Aldrich/Merck Life Sciences, Madrid, Spain), as previously described [28]. Initially, 5 mL of blood collected in an EDTA tube was diluted 1:1 with a 0.9% saline solution. Then, 3 mL of Histopaque 1077 was added to this mixture. The combined mixture was subsequently centrifuged for 30 min at 2000 rpm without braking. After centrifugation, the PBMC layer was carefully collected and washed three times with saline solution. Following the final centrifugation, the supernatant was removed, and the PBMCs were resuspended in 5 mL of Gibco AIM-V medium (Fisher Scientific Spain, Madrid, Spain). Cell viability was assessed by mixing 20 µL of the PBMC suspension with 20 µL of trypan blue (Fisher Scientific), and live cells were counted using a TC20 automated cell counter (Bio-Rad, Barcelona, Spain). Only samples with at least 70% viable cells were retained for co-culture with postbiotics. Finally, the PBMC suspension was adjusted to a concentration of 1 million cells/mL in Gibco AIM-V medium.

### 2.3. Postbiotic Stimulation

Dog PBMCs were cultured with each one of the two heat-killed (Tyndallized) *Lactobacillus rhamnosus SGL01* or *Lactobacillus reuteri SGL06* strains that are included in the tablets and spray of the Linkskin product line (Nextmune, Palazzo Pignano, Cremona, Italy); cells cultured in saline without postbiotics served as the negative control. The PBMC-to-bacteria ratio was 1:5 (for instance, 2 × 10^5^ PBMCs and 1 × 10^6^ Tyndallized bacteria), as previously described [29]. Cells were cultured for 72 h, and supernatants were collected both before incubation and after 12, 24, 48, and 72 h from the start of co-culture with the postbiotics.

### 2.4. Cytokine Measurement

For each culture condition and time point, cytokine levels were quantified using ELISA (Bio-Techne R&D Systems, Minneapolis, MN, USA), which is validated for measuring canine IFN-γ, IL-4, IL-10, and IL-12. After determining the cytokine concentrations in the supernatant, the stimulation indices (SI) of the cytokine levels were calculated from the cytokine levels after culture with each of the two tested Tyndallized bacteria compared to those cultured with saline at the same time point.

### 2.5. Statistical Analyses

The cytokine levels and SIs were compared using nonparametric repeated-measures ANOVA (Friedman test), with post-hoc tests assessing each time point against unstimulated cells at Time 0 (cytokine levels) or 12 h (SIs). Analyses were performed using Prism version 10.3.1 (GraphPad Software, Boston, MA, USA).

## 3. Results

### 3.1. Cytokine Profile of Healthy Dog PBMCs After Postbiotic Stimulation

To evaluate the immunomodulatory effects of Tyndallized *L. rhamnosus* and *L. reuteri*, PBMCs were collected from healthy dogs and cultured separately with each of these two species for 72 h. Interferon-γ and IL-12 levels were measured to assess the postbiotics’ impact on Th1 polarization, while those of IL-4 and IL-10 were selected as biomarkers for Th2 and Treg polarization, respectively.

**Figure 1:**
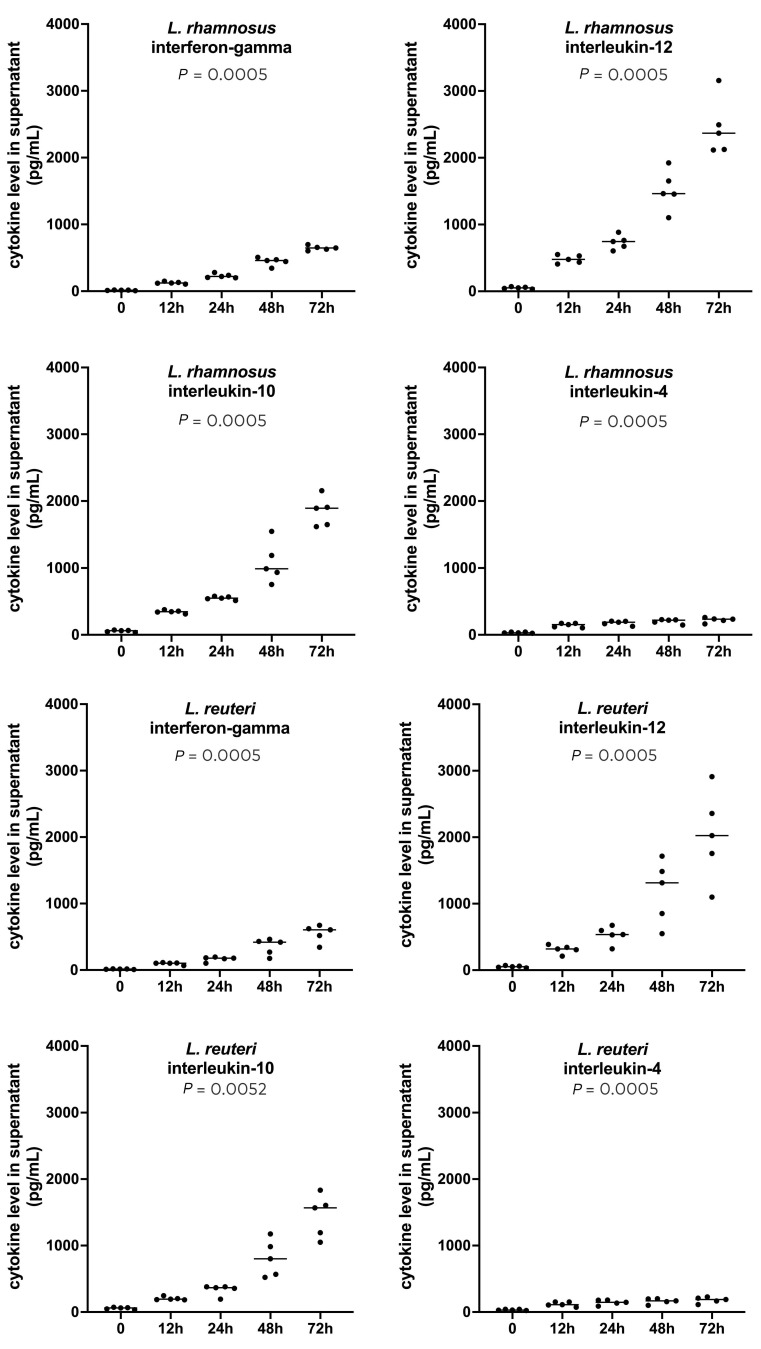
Cytokine concentrations (in pg/mL) of IFN-γ, IL-12, IL-10, and IL-4 in the supernatants of normal canine PBMCs before and during 72 h of incubation with Tyndallized *L. rhamnosus* and *L. reuteri*. The *p* values reported here are those from the Friedman nonparametric repeated-measure ANOVA.

There was no change over time in the production of cytokines in cells cultured with saline (Table S1). In contrast, the incubation of canine PBMCs with either of the two Tyndallized lactobacilli resulted in significant increases (Friedman nonparametric repeated ANOVA) in the cytokines released in the culture supernatants, beginning at 12 h after the start of the incubation period and continuing throughout the 72 h of co-culture (Figure 1, Table S1). For both lactobacilli and all four cytokines, the levels were significantly higher after 48 and 72 h compared to those at baseline (Dunn’s multiple comparison tests).

While the levels of the four cytokines increased after co-culture with either of the two postbiotics, the magnitude of that rise was variable. After 72 h of incubation, IL-12 and IL-10 showed the highest levels of release at approximately 2000–2500 pg/mL and 1500–2000 pg/mL, respectively. The amount of IFN-γ secreted was more moderate (500–700 pg/mL at 72 h), while that of IL-4 was markedly lower (around 200 pg/mL at 72 h). The median (inclusive range) IL-12 to IL-4 ratios for the five dogs were 11 (9–14) and 10 (9–17) for *L. rhamnosus* and *L. reuteri*, respectively, suggesting that the produced cytokines would favor a Th1 rather than a Th2 immune response.

### 3.2. Stimulation Indices

**Figure 2:**
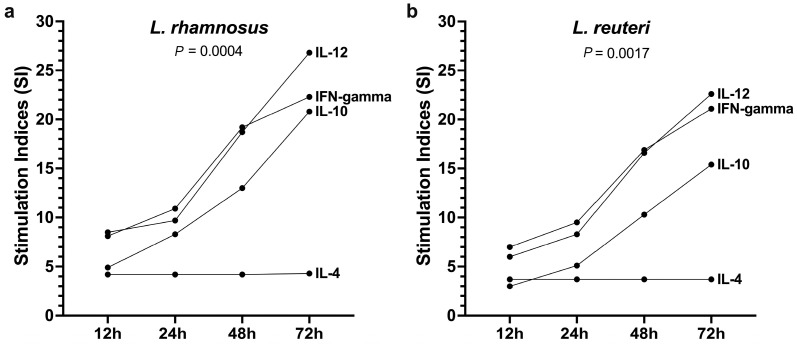
Median stimulation indices of cytokines released in the supernatants of healthy canine PBMCs after 12 to 72 h of incubation with (**a**) Tyndallized *L. rhamnosus* and (**b**) Tyndallized *L. reuteri*. Stimulation indices were calculated from the cytokine levels after culture with each of the two tested Tyndallized bacteria compared to those cultured with saline at the same time point. The *p* values reported here are those from the Friedman nonparametric repeated-measure ANOVA.

We compared the stimulation indices (SI) of cytokines released after the culture of PBMCs with Tyndallized lactobacilli to the results for those incubated with saline (the unstimulated PBMCs). The two postbiotics demonstrated similar stimulation effects, as shown in Figure 2. The stimulation index for both types of bacteria progressively and significantly (Friedman nonparametric repeated-measure ANOVA) increased over time. At the 72h mark, the stimulation of IL-12, IFN-γ, and IL-10 production was relatively similar, with an SI ranging from 15 to 30. In contrast, the stimulation of IL-4 remained low throughout the duration of the study period (Figure 2). Significant differences in the SI for all cytokines were observed only after 72 h of culture when compared to those at 12 h (Dunn’s multiple comparison tests; *p* = 0.0078 for *L. rhamnosus* and *p* = 0.0411 for *L. reuteri*).

Altogether, these results indicate that Tyndallized *Lactobacillus rhamnosus* and *reuteri* can stimulate healthy canine PBMCs to promote the release of Th1 (IL-12 and IFN-γ) and Treg (IL-10) cytokines, in contrast to the much lower stimulation levels of the Th2 cytokine IL-4.

## 4. Discussion

In this simple co-culture experiment, we demonstrated for the first time that the two *Lactobacillus* species contained in the Linkskin product line, *L. rhamnosus* and *L. reuteri*, in an inactive state following Tyndallization, exhibited immunomodulating effects on healthy canine PBMCs in vitro. Heat-killed *L. rhamnosus* and *L. reuteri* stimulated the secretion of the Th1 cytokines IL-12 and IFN-γ, as well as that of the Treg cytokine IL-10, while the effect on the Th2 cytokine IL-4 was more subdued.

These results align with findings from previous studies using other heat-killed *Lactobacillus* strains, which have demonstrated a tendency to skew immune responses toward Th1 polarization in cells from various mammalian species. For example, heat-killed *Lactobacillus casei* Shirota stimulated the murine monocyte/macrophage cell line J774A to produce the Th1 cytokines IL-12 and TNF-α and the Treg cytokine IL-10 [30]. In another experiment, heat-killed lactobacilli also promoted the secretion of IL-12, IFN-γ, and IL-10 in mouse splenocytes; the effects on dendritic cells were, however, different, with a similar increase in IL-12 production, without a change in IL-10 secretion [31].

The results of our postbiotic stimulation of canine PBMCs also suggested the induction of a Th1 cytokine profile, since IFN-γ and IL-12 are considered typical representatives of such a response. In acute stages of canine AD, the cytokine balance appears to favor Th2 cytokines [4]. Therefore, promoting the secretion of Th1 cytokines with postbiotics at the expense of those that encourage Th2 responses could be beneficial. Allergen-specific immunotherapy, which could be seen as one of the most effective long-term strategies to prevent flares of canine AD, has been shown to induce a similar cytokine shift toward Th1 by enhancing IFN-γ expression [32].

In this study, using PBMCs isolated from healthy dogs, we demonstrated a *Lactobacillus*-induced stimulation of IL-10 secretion, a cytokine indicative of Treg polarization. Regulatory T cells are considered to play a crucial role in controlling Th2 responses to allergens [33]. Indeed, a study performed in a murine model of AD demonstrated that a decreased FOXP3+ expression, a transcription factor reflecting Treg activity, was correlated with an increased inflammatory response, with elevated Th2 cytokines and IgE production [34]. By depleting these regulatory T cells, the authors showed their importance in controlling atopic inflammation [34]. In dogs, the few studies regarding the role of IL-10 have led to more equivocal results (reviewed in [3]).

In this study, we concentrated solely on the effects of Tyndallized bacteria because utilizing non-viable postbiotics offers distinct advantages. Firstly, since these bacteria are no longer alive, they have a longer shelf life. Maintaining stable live microorganisms can be difficult, and the ratio of live to dead bacteria tends to decrease over time in probiotic-containing products [23]. In their recent consensus statement, Salminen et al. raised the relevant question of determining the contribution of inanimate versus live microorganisms in therapeutic studies performed with probiotics [23]. While the intrinsic stability of postbiotics is attractive, they are considered safer than probiotics because they have lost their ability to proliferate and compete with local microbiota [23]. Indeed, safety concerns have been raised when using probiotics in vulnerable populations [35]. Although extremely rare, live probiotics can translocate from the gut to the bloodstream and cause bacteremia [36].

In our study, we sampled peripheral blood mononuclear cells (PBMCs) from healthy dogs and cultured them in the presence of postbiotics. Further research is needed to determine if similar results can be obtained in vitro using PBMCs isolated from atopic dogs. However, we suspect that the findings in the latter population would be comparable to those obtained with healthy dogs. Indeed, a study was conducted using PBMCs from human patients with grasspollen allergies alongside those from non-allergic individuals [37]. In that study, the cells were co-cultured with grass pollen allergens and either inactivated *Lactobacillus acidophilus* or a non-pathogenic strain of *Escherichia coli* (Nissle). The results indicated that both bacteria modulated the allergic immune response triggered by the allergen by stimulating a similar Th1 response in cells from both healthy and allergic patients [37]. Of additional interest would be the culture of PBMCs from atopic dogs with allergens and postbiotics, as these conditions would better emulate what occurs in an allergic patient.

The therapeutic potential of heat-killed lactobacilli has been studied in various mammal species, even though few studies specifically evaluated the two species examined here. While *L. rhamnosus* remains the most studied postbiotic in clinical settings, there are few studies available for review.

In the murine NC/NgA model of atopic dermatitis (AD), the daily oral administration of Tyndallized *L. rhamnosus* IDCC 3201 for 8 weeks resulted in a significant reduction in both dermatitis scores and scratching frequencies compared to those of mice treated with saline. Additionally, a moderate but significant decrease in total IgE levels was observed in mice treated with the postbiotic compared to those receiving saline. Surprisingly, the levels of IL-4, IFN-γ, IL-12, and IL-10 in cultures from lymph node cells showed concentration-dependent reductions. As a result, no measurable change was observed in the IFN-γ/IL-4 ratios among the different treatment groups [38]. Such a reduction in cytokine levels in cultures of lymph node cells is not surprising, as Won et al. previously demonstrated that serum IFN-γ increased after probiotic administration, while at the same time, it decreased in lymph node cells [39]; these dual effects illustrate the complexity of the immune regulation induced by probiotics.

In a randomized controlled trial, Tyndallized *L. rhamnosus* was used for the reactive treatment of children with atopic diseases. There was a significant reduction in SCORAD lesion scores for patients treated with the postbiotic compared to those receiving a placebo. However, the small difference seen in the lesion scores (24.25 vs. 28.13) raises the question of the clinical relevance of these results [40]. Similarly, a meta-analysis of three trials confirmed that the oral administration of *Lactobacillus* postbiotics leads to a significant, yet small, difference in mean SCORAD scores [41].

Currently, there is only one clinical study that evaluates the effectiveness of Tyndallized bacteria—although not specifically lactobacilli—in reducing symptoms in dogs with AD. In this study, dogs were treated for 12 weeks with heat-killed *Enterococcus faecalis* FK-23. The results showed a moderate but statistically significant reduction in the CADESI-4 skin lesion score, which decreased from 25.6 to 17.8, indicating a residual—or incompletely controlled—mild AD; there was no significant effect on pruritus or daily medication scores, however. Finally, the treatment response was evaluated as being more favorable in dogs receiving postbiotics than in those treated with placebo [42].

Even though further research is needed to firmly establish the cytokine and T cell dynamics in the skin of dogs with AD, as contradictory results exist on this topic [3], modulating the T cell immune response with postbiotics should—at least theoretically—provide a cost-effective anti-allergic strategy. Given that postbiotics exhibit a lower immunomodulating potency compared to that of currently registered anti-allergic drugs, research should focus on their use as adjuvants during reactive therapy. This approach may allow for a reduced dosage of existing medications, while still maintaining full control over the symptoms. Alternatively, postbiotics could be explored as a means of helping to prevent flares of itch and skin lesions in atopic dogs that have had their symptoms initially managed with standard pharmacotherapy. Such a proactive therapy with postbiotics would represent a form of allergen-independent “nonspecific” immunotherapy.

## 5. Conclusions

This preliminary study underscores the immunomodulating properties of Linkskin’s heat-killed *Lactobacillus rhamnosus* and *L. reuteri* on PBMCs collected from healthy dogs. The examined postbiotics stimulated the in vitro secretion of Th1 cytokines, specifically IL-12 and IFN-γ, as well as the regulatory T cell cytokine IL-10. In contrast, their effect on the Th2 cytokine IL-4 was less pronounced. Overall, the stimulated cytokine production profile is expected to encourage a Th1 response, which may yield anti-allergic effects. These early findings suggest that the evaluated postbiotics merit further assessment for their clinical benefits as adjuvants for reactive treatment or as nonspecific immunotherapies for preventing relapses of allergic flares in atopic dogs.

## Data Availability

Raw data are available in Table S1.

## Supplementary Materials

The following are available online at https://www.mdpi.com/article/10.3390/vetsci12030226/s1. Table S1: cytokine levels and stimulation indices.
