# Supplementary material for: Immunomodulating Effects of Heat-Killed Lactobacillus rhamnosus and Lactobacillus reuteri on Peripheral Blood Mononuclear Cells from Healthy Dogs
Source: Vet Sci. 2025 Mar 2;12(3):226. doi: 10.3390/vetsci12030226 (PMC11946048; doi:10.3390/vetsci12030226)
Supplement: Supplementary file 1 [file vetsci-12-00226-s001.zip › vetsci-3474775-supplementary.pdf]

Supplementary Material: Table S1: cytokine levels and stimulation indices (SI)

		pg/mL					Stimulation Indices (SI)					
		Dog 1	Dog 2	Dog 3	Dog 4	Dog 5	Dog 1	Dog 2	Dog 3	Dog 4	Dog 5	median
**IL12 T12**	*L. Rhamnosus*	529,9	550,0	408,0	477,4	436,0	7,5	12,6	12,3	8,0	8,5	**8,5**
	L. Reuteri	387,0	318,8	210,9	342,5	307,3	5,5	7,3	6,3	5,7	6,0	**6,0**
	Placebo	70,6	43,5	33,3	59,9	51,3	X	X	X	X	X	
**IL12 T24**	*L. Rhamnosus*	763,1	883,3	746,0	674,6	605,4	9,1	14,6	16,6	9,4	9,7	**9,7**
	L. Reuteri	674,3	531,0	321,2	596,7	535,8	8,0	8,8	7,1	8,3	8,6	**8,3**
	Placebo	84,0	60,6	44,9	71,9	62,2	X	X	X	X	X	
**IL12 T48**	*L. Rhamnosus*	1923,3	1463,3	1103,0	1652,7	1456,8	18,6	20,4	19,1	18,6	18,7	**18,7**
	L. Reuteri	1715,0	851,4	548,4	1484,5	1314,3	16,6	11,9	9,5	16,7	16,9	**16,6**
	Placebo	103,2	71,7	57,6	89,1	77,8	X	X	X	X	X	
**IL12 T72**	*L. Rhamnosus*	3155,9	2368,3	2115,5	2493,3	2124,9	26,8	26,9	29,4	24,4	23,7	**26,8**
	L. Reuteri	2910,8	1755,3	1099,4	2357,9	2024,0	24,7	19,9	15,3	23,1	22,6	**22,6**
	Placebo	117,8	88,0	72,1	102,2	89,7	X	X	X	X	X	

		pg/mL					Stimulation Indices (SI)					
		Dog 1	Dog 2	Dog 3	Dog 4	Dog 5	Dog 1	Dog 2	Dog 3	Dog 4	Dog 5	median
**IL10 T12**	*L. Rhamnosus*	354,6	376,4	312,3	346,0	341,5	4,90	5,38	5,31	2,95	3,02	**4,9**
	L. Reuteri	201,4	245,6	183,5	194,3	186,3	2,79	5,38	5,31	2,95	3,02	**3,0**
	Placebo	72,3	45,7	34,6	65,8	61,7	X	X	X	X	X	
**IL10 T24**	*L. Rhamnosus*	567,37	577,78	513,45	548,76	541,32	6,40	8,98	10,98	7,85	8,27	**8,3**
	L. Reuteri	379,87	365,77	194,56	355,66	380,56	4,28	5,69	4,16	5,09	5,82	**5,1**
	Placebo	88,67	64,32	46,78	69,88	65,44	X	X	X	X	X	
**IL10 T48**	*L. Rhamnosus*	1546,0	988,8	754,6	1188,5	935,4	16,49	14,02	11,87	13,00	12,01	**13,0**
	L. Reuteri	1174,6	567,9	521,2	984,2	801,3	12,52	8,05	8,20	10,76	10,29	**10,3**
	Placebo	93,8	70,5	63,6	91,5	77,9	X	X	X	X	X	
**IL10 T72**	*L. Rhamnosus*	2154,3	1893,3	1616,8	1905,4	1647,5	21,14	20,83	22,49	19,29	17,78	**20,8**
	L. Reuteri	1567,3	1193,6	1050,7	1832,5	1605,2	15,38	13,13	14,61	18,55	17,32	**15,4**
	Placebo	101,9	90,9	71,9	98,8	92,7	X	X	X	X	X	

		pg/mL					Stimulation Indices (SI)					
		Dog 1	Dog 2	Dog 3	Dog 4	Dog 5	Dog 1	Dog 2	Dog 3	Dog 4	Dog 5	median
**IFNg T12**	*L. Rhamnosus*	130,9	150,2	105,5	121,9	118,6	7,5	13,5	13,7	7,9	8,1	**8,1**
	L. Reuteri	112,9	102,2	66,8	105,0	102,2	6,5	9,2	8,7	6,8	7,0	**7,0**
	Placebo	17,4	11,1	7,7	15,4	14,7	X	X	X	X	X	
**IFNg T24**	*L. Rhamnosus*	221,2	279,2	237,5	206,2	200,8	10,3	16,5	20,4	10,7	10,9	**10,9**
	L. Reuteri	196,2	170,4	103,0	182,9	178,1	9,1	10,1	8,9	9,5	9,7	**9,5**
	Placebo	21,5	16,9	11,6	19,3	18,5	X	X	X	X	X	
**IFNg T48**	*L. Rhamnosus*	507,2	444,2	344,0	471,5	458,8	18,5	21,6	21,7	19,0	19,2	**19,2**
	L. Reuteri	462,2	269,6	175,9	430,0	418,5	16,9	13,1	11,1	17,3	17,5	**16,9**
	Placebo	27,4	20,6	15,9	24,8	23,9	X	X	X	X	X	
**IFNg T72**	*L. Rhamnosus*	698,5	655,0	602,9	646,6	628,4	21,9	25,1	29,1	22,3	22,0	**22,3**
	L. Reuteri	672,0	519,2	342,9	622,5	605,1	21,1	19,9	16,6	21,5	21,2	**21,1**
	Placebo	31,9	26,0	20,7	29,0	28,5	X	X	X	X	X	

		pg/mL					Stimulation Indices (SI)					
		Dog 1	Dog 2	Dog 3	Dog 4	Dog 5	Dog 1	Dog 2	Dog 3	Dog 4	Dog 5	median
**IL4 T12**	*L. Rhamnosus*	119,5	153,6	104,5	171,9	172,3	4,0	5,6	4,6	4,2	4,2	**4,2**
	L. Reuteri	105,4	110,6	72,4	151,4	151,7	3,6	4,0	3,2	3,7	3,7	**3,7**
	Placebo	29,6	27,7	22,7	41,4	41,4	X	X	X	X	X	
**IL4 T24**	*L. Rhamnosus*	167,1	188,4	128,5	203,9	202,2	4,1	5,7	4,7	4,2	4,2	**4,2**
	L. Reuteri	147,2	135,3	88,8	179,4	177,9	3,7	4,1	3,2	3,7	3,7	**3,7**
	Placebo	40,3	33,2	27,4	48,5	48,1	X	X	X	X	X	
**IL4 T48**	*L. Rhamnosus*	218,5	191,1	145,8	224,0	227,7	5,7	4,2	4,8	4,2	4,2	**4,2**
	L. Reuteri	156,6	168,2	100,5	197,0	200,3	4,1	3,7	3,3	3,7	3,7	**3,7**
	Placebo	38,0	45,6	30,7	52,9	53,7	X	X	X	X	X	
**IL4 T72**	*L. Rhamnosus*	233,7	215,3	163,2	259,8	237,2	5,8	4,2	4,8	4,3	4,2	**4,3**
	L. Reuteri	167,3	189,4	112,3	228,3	208,5	4,1	3,7	3,3	3,8	3,7	**3,7**
	Placebo	40,4	51,0	34,0	60,8	55,8	X	X	X	X	X	
